# Shrub Expansion Impacts on Carbon, Nitrogen, and Sulfur Cycles and Microorganism Communities in Wetlands in Northeastern China

**DOI:** 10.3390/microorganisms13092014

**Published:** 2025-08-28

**Authors:** Shenzheng Wang, Lin Li, Xiaoyu Fu, Haixiu Zhong, Rongtao Zhang, Xin Sui

**Affiliations:** 1 Heilongjiang Provincial Key Laboratory of Ecological Restoration and Resource Utilization for Cold Region, School of Life Sciences, Heilongjiang University, Harbin 150090, China; wangshenzheng2000@163.com; 2 Heilongjiang Ecology Institute, Harbin 150081, China; lilin_1002@163.com; 3 Institution of Nature and Ecology, Heilongjiang Academy of Sciences, Harbin 150040, China; 18646583130@163.com (X.F.); zhx971030@163.com (H.Z.)

**Keywords:** element cycle, metagenome, shrub expansion, soil microorganism, wetland

## Abstract

Marsh wetland degradation and shrub expansion, driven by human activities and climate change, can impact carbon, nitrogen, and sulfur cycles by soil microorganisms. There is a paucity of systematic and in-depth research on the impact of shrub expansion in temperate wetlands on soil element cycles, which is a pressing scientific issue that demands resolution. This study used metagenomic sequencing and soil analysis methods to investigate the impact of shrub expansion in the Sanjiang Plain wetlands on carbon, nitrogen, and sulfur cycles in temperate wetland soils, as well as on functional microbial communities. Shrub expansion significantly altered soil carbon, nitrogen, and sulfur cycle processes and the composition (β diversity) of associated functional microbial communities, despite minimal changes in overall α diversity. Significant shifts occurred in the abundance of cycle pathways and related functional genes. Ammonia nitrogen, moisture, and total phosphorus were identified as the primary factors influencing these cycles and the functional microbial communities. Changes in the abundance of specific cycling pathways following shrub expansion are key drivers of functional community structure transformation. These changes may significantly reduce the long-term carbon sequestration potential of wetlands and affect regional climate feedback by altering greenhouse gas fluxes. The findings provide a theoretical basis for managing shrub expansion and assessing wetland function.

## 1. Introduction

As a crucial type of wetland ecosystem, marsh wetlands possess a unique ecosystem structure, complex and diverse habitats, and rich biodiversity, playing a vital role in the biogeochemical cycles of ecosystems [1,2]. They not only provide highly efficient water purification and climate regulation functions and serve as critical habitats and breeding grounds for numerous rare species but also exhibit complex and subtle characteristics within ecological cycles systems [3,4]. Located in the eastern Heilongjiang Province, the Sanjiang Plain represents the largest concentration of freshwater wetlands in China and serves as a significant ecological barrier in Northeast Asia [5,6]. This region is not only a core agricultural production base ensuring national food security but also holds irreplaceable ecological value in maintaining global biogeochemical cycles, biodiversity conservation, hydrological regulation, and nutrient balance [7,8,9,10]. Furthermore, the Sanjiang Plain wetlands constitute the largest wetland area in East Asia outside of Siberia. However, with the continuously increasing intensity of human activities (such as agricultural reclamation and artificial drainage) and the ongoing impacts of climate change, the wetlands of the Sanjiang Plain have undergone degradation. Vegetation composition has progressively shifted, and declining water tables have led to the expansion of drought-tolerant shrub species, resulting in the formation of a shrub-herb patchy mosaic pattern. This process directly alters the patterns of exogenous carbon input and exerts potential effects on soil microbial-mediated carbon, nitrogen, and sulfur cycle processes within the wetlands. Notably, previous studies have primarily focused on the impact of shrub expansion on microbial biomass and diversity [11,12,13], while its regulatory mechanisms on microbial community functionality remain unclear.

Soil microbial communities play a pivotal role in the functioning of terrestrial ecosystems, participating in key processes such as biogeochemical cycles, energy flow, detritus decomposition, and climate regulation [14,15,16]. Moreover, by regulating organic matter decomposition, redox reactions, and coupled electron transfer chains, they dominate the ecological functions of wetlands [17]. Abundant research indicates that shrub expansion affects soil microbial abundance, community structure, and composition through alterations in soil physicochemical properties [18,19]. Shrub encroachment can disrupt the water-heat balance, resource allocation, and microclimate regulation mechanisms of wetland systems, thereby triggering changes in soil microbial community structure [20,21].

As is well established, soil microbial communities are highly sensitive to environmental changes. Although microbial biomass constitutes only a small fraction of soil organic matter, it significantly drives the cycles of elements such as carbon, nitrogen, and sulfur at the ecosystem scale [22]. It has been reported that shrub expansion generally enhances plant productivity and diversity in arid and semi-arid regions, leading to increased soil organic carbon storage (SOCS) and soil total nitrogen storage (STNS) [23,24]. Nevertheless, research on how shrub expansion affects soil carbon, nitrogen, and sulfur cycles has predominantly concentrated on arid regions, Arctic tundra, grasslands, etc. [25,26,27]. In contrast, systematic and in-depth studies on how shrub expansion in temperate wetlands influences soil elemental cycles are still lacking, constituting a significant scientific question that urgently needs to be addressed. In our previous studies, we found that shrub expansion altered soil microbial abundance, community structure, and composition in the Sanjiang Plain wetlands and modified methane emissions through a synergistic pathway involving soil physicochemical properties and oxidation-related genes. Based on these findings, we infer that the soil carbon, nitrogen, and sulfur cycles and their associated functional microbial communities in the Sanjiang Plain wetlands undergo changes influenced by shrub expansion.

Based on the aforementioned background, the current study was conducted at the field experimental station of the Honghe National Nature Reserve, Heilongjiang Provincial Academy of Sciences. Focusing on varying degrees of shrub expansion (CK, SI, MI, HI), we employed metagenomic sequencing and soil physicochemical analysis to reveal the impact of shrub expansion on soil carbon, nitrogen, and sulfur cycles and their associated functional microorganisms in temperate wetlands. Specifically, the objectives of our research were to determine how shrub expansion (1) effects soil carbon, nitrogen, and sulfur cycle pathways, (2) alters the structure and composition of related functional microbial communities in the Sanjiang Plain wetlands, and (3) regulates these changes through particular pathways.

## 2. Materials and Methods

### 2.1. Study Site

**Figure 1:**
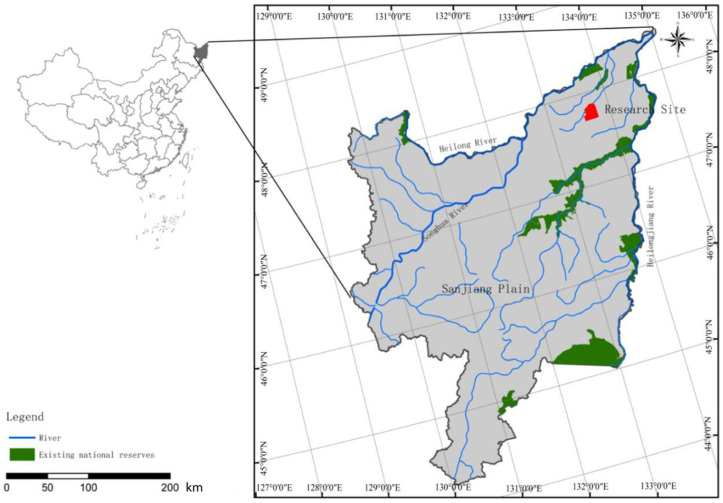
The red area indicates the experimental site: the Honghe National Nature Reserve in the Sanjiang Plain of Northeast China. We conducted experiments here to study the impact of shrub expansion on the elemental cycle in wetlands.

The research was conducted at the field experimental station of the Heilongjiang Provincial Academy of Sciences, situated within the Honghe National Nature Reserve (47°42′–47°52′ N, 133°34′38″–133°46′29″ E) in the Sanjiang Plain of Northeast China (Figure 1). The study site experiences a typical temperate humid/semi-humid monsoon climate. The mean annual temperature is 1.9 °C, with an average annual precipitation of 585 mm and evaporation of 1166 mm [28], with 50–70% concentrated in the summer. The wetland area covers approximately 21,800 hm^2^, primarily dominated by Calamagrostis angustifolia wetlands [29]. The geomorphology within the reserve is characterized by a saucer-shaped depression, the most common landform for marsh development in the Sanjiang Plain. The soils are classified as typical albic hydric soil and fibric organic soil. The main vegetation includes *Calamagrostis angustifolia*, *Glyceria spiculosa*, *Carex lasiocarpa*, and *Carex pseudocuraica*, among others.

### 2.2. Experimental Design

**Figure 2:**
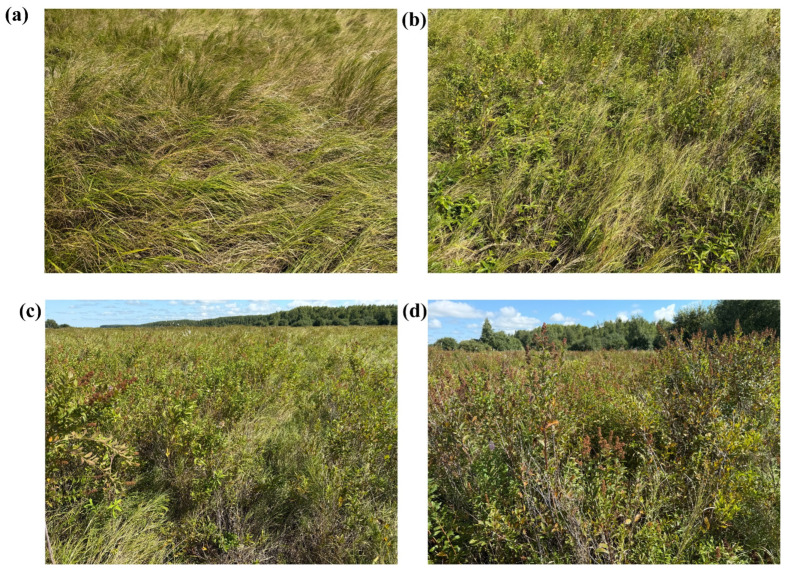
Photographs of plots with different levels of shrub encroachment. (**a**) no expansion; (**b**) mild expansion; (**c**) moderate expansion; (**d**) severe expansion.

In July 2022, the experiment selected wetlands with different levels of shrub expansion intensity, all of which had been invaded by the representative shrub, saltbush. The classification of shrub expansion intensity plots was conducted on the basis of shrub coverage (a) within the marshland (the ratio of the area covered by shrub crowns to the total area) (Figure 2). The following categorization was employed: when a = 0 in the plot, it was designated as no expansion (CK); when 0 < a ≤ 30%, it was classified as mild expansion (SI); when 30% < a ≤ 70%, it was designated as moderate expansion (MI); and when 70% < a ≤ 100%, it was classified as severe expansion (HI). A total of three replicate plots were selected for each shrub expansion level, thus resulting in 12 marsh plots exhibiting a range of shrub expansion types.

### 2.3. Soil Sample Collection and Analysis

In 2022, surface soil (approximately 0–20 cm) was collected from each plot using a 5 cm inner diameter drill. Five to ten soil samples were collected from each plot at random. Subsequently, soil samples from each plot were amalgamated, placed in sealed bags, stored at 4 °C, and immediately transported to the laboratory. Soil samples were subjected to homogenization using a 2 mm sieve, after which they were divided into two distinct parts for separate analyses. One part was stored at −80 °C for the purpose of conducting a microbial community analysis, while the other part was subjected to air-drying to facilitate the determination of the soil’s physical and chemical properties.

### 2.4. Determination of Soil Physical and Chemical Properties

The soil physicochemical properties were determined in accordance with the methodology outlined in our previous study [30]. Briefly, the soil pH was measured using a pH meter and soil-to-water ratio of 1:2.5 *w*/*v*. Soil organic carbon and total nitrogen were measured using an elemental analyzer (Elementar, Langenselbold, Germany). Ammonia nitrogen was examined with a continuous flow analysis (SAN++, Skalar Analytical, Harbin, China). Soil moisture content measured gravimetrically. Total phosphorus was measured with a spectrophotometer. Available phosphorus was measured using a colorimetric method upon extraction with 0.5 M NaHCO_3_. The determination of ammonia nitrogen (AN) uses the HCl hydrolysis-diffusion method.

### 2.5. DNA Extraction, Metagenome Sequencing, and Data Processing

Total genomic DNA was extracted from soil samples using the ALFA-SEQ Advanced Soil DNA Kit (ALFA-SEQ, Harbin, China, v1.1) according to the manufacturer’s instructions. DNA integrity and potential contamination were assessed via 1% agarose gel electrophoresis, while the concentrations and purity (A260/A280 and A260/A230 ratios) were measured using a Qubit 3.0 Fluorometer and a Nanodrop One Spectrophotometer (Thermo Fisher Scientific, Harbin, China). The sequencing libraries were prepared using the ALFA-SEQ DNA Library Prep Kit, with the quality control conducted using a Qubit 4.0 Fluorometer (Life Technologies, Harbin, China) and a Qsep400 Nucleic Acid Analyzer (Houze Biotech, Harbin, China). High-throughput paired-end sequencing (2 × 150 bp) was performed using an Illumina NovaSeq 6000 platform [31].

Raw sequencing reads were quality-trimmed, and adapter sequences were removed using Trimmomatic v0.36. De novo assembly of the filtered reads was performed using MEGAHIT v1.0.6, generating scaffolds (scaftigs ≥ 500 bp) from both individual and pooled datasets [32]. Open reading frames (ORFs) were predicted from the assembled scaftigs using MetaGeneMark v3.38. Redundancy was minimized using CD-HIT v4.7, with a sequence identity threshold of 95% and coverage cutoff of 90%, resulting in a non-redundant UniGene catalog, where the representative sequences were selected based on length [33]. Gene abundance profiles across samples were calculated by aligning clean reads to the UniGene catalog using BBMAP v38.87.

UniGene sequences were taxonomically annotated by aligning them against the NCBI NR database using BLASTP (E-value ≤ 1 × 10^−10^). Hierarchical classification was assigned using the Lowest Common Ancestor (LCA) algorithm implemented in MEGAN version 6.24 [34]. Functional annotation was performed through homology searches against the Kyoto Encyclopedia of Genes and Genomes (KEGG) database using DIAMOND version 2.1.8 [35].

### 2.6. Statistical Analysis

Microbial community differences were assessed using diversity indices, including the Shannon, Chao1, and Heip indices, along with non-metric multidimensional scaling (NMDS). Microbial community composition was visualized based on the relative abundance at various taxonomic levels. CH4 metabolic pathways were analyzed by aligning UniGenes to the KEGG database [35] using DIAMOND [36]. Gene abundances normalized by RPKM were aggregated into pathway-level relative abundances. Differential pathway abundances were identified using DESeq2 version 1.44.0 [37] with FDR correction. KEGG Mapper and ggplot2 were used to visualize the pathway maps and annotate the fold-change data. One-way ANOVA was conducted using IBM SPSS Statistics (version 26.0; IBM Corp., Armonk, NY, USA, 2019), and all graphs were generated using OriginPro (version 2023; OriginLab Corp., Northampton, MA, USA, 2023). Data preprocessing was performed using readxl (v1.4.3; Wickham & Bryan 2023 [38]) and glue (v1.6.2; Hester 2023 [39]) packages. Given the potential non-normality inherent in microbial community data, bootstrap tests (*n* = 200) were employed to verify statistical significance (α = 0.05).

## 3. Results

### 3.1. Impact of Shrub Expansion on the Physicochemical Properties of Soils

The results indicated that shrub expansion significantly increased AN content while reducing soil moisture (Table 1). Soil moisture content is significantly higher in areas with no expansion and slight expansion than in areas with severe expansion, but it is still significantly lower than in areas with no expansion. In contrast, short-term shrub expansion did not significantly alter other soil physicochemical properties, including PH, TN, TOC, TP, and AP.

### 3.2. Changes in Microbial Diversity Related to Carbon, Nitrogen, and Sulfur Cycles

**Figure 3:**
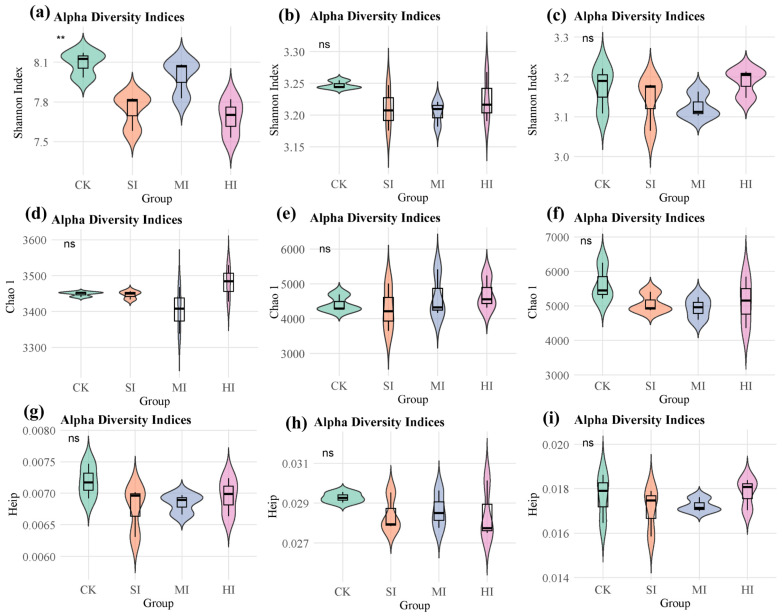
α-Diversity of cycle-related microbial communities under different levels of shrub expansion: (**a**,**d**,**g**) C cycles; (**b**,**e**,**h**) N cycles; (**c**,**f**,**i**) S cycles. ** indicates differences at the 0.01 level of significance. ns indicates no significant difference. CK: no expansion; SI: mild expansion; MI: moderate expansion; HI: severe expansion.

The results demonstrated that there were no significant differences in the Chao 1 index and Heip index of microbial communities performing various cycle functions under different levels of shrub expansion (Figure 3d–i). However, the Shannon index of microorganisms performing carbon cycle functions changed significantly under different shrub expansion conditions. Under no expansion and moderate expansion levels, the Shannon index of microorganisms performing carbon cycle functions was significantly higher than that under mild expansion and severe expansion, while the Shannon index of microbial communities performing nitrogen and sulfur cycle functions remained unchanged (Figure 3a–c).

**Figure 4:**
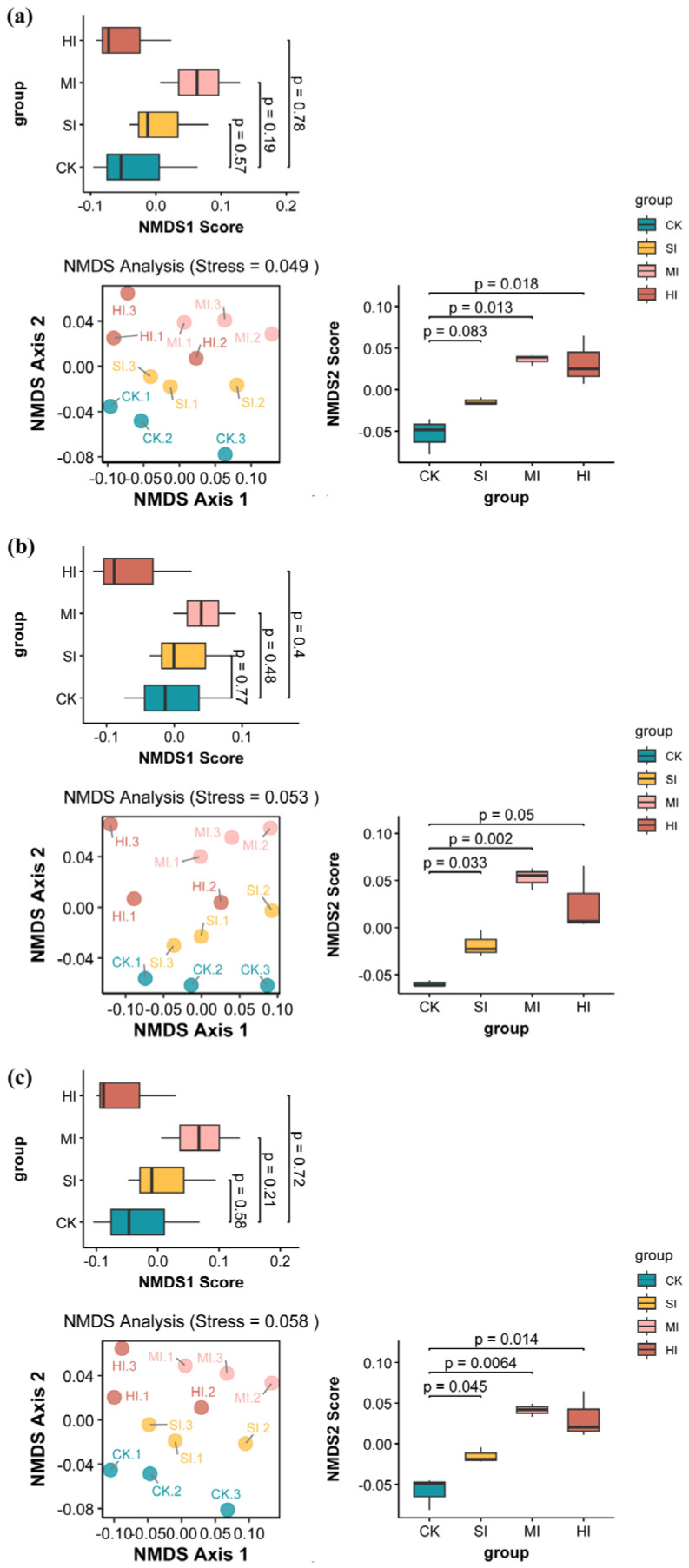
NMDS analysis and marginal box plots of microbial communities associated with the C cycle (**a**), N cycle (**b**), and S cycle (**c**) under different levels of shrub expansion. CK: no expansion; SI: mild expansion; MI: moderate expansion; HI: severe expansion.

As demonstrated in Figure 4, the expansion of shrubbery had a substantial impact on the β-diversity of microbial communities associated with diverse cycle functions. Soil microbial communities are significantly separated by different degrees of shrub expansion. Figure 4a: The NMDS analysis of carbon cycle-related microbial communities revealed significant differences between mild expansion and no expansion (*p* = 0.013) and severe expansion and no expansion (*p* = 0.018) in the NMDS Score 2 margin box plot. As illustrated in Figure 4b, the NMDS analysis of the nitrogen cycle functional microbial community reveals statistically significant disparities between moderate expansion and no expansion (*p* = 0.033) and mild expansion and no expansion (*p* = 0.002) as depicted in the NMDS Score 2 marginal boxplot. Figure 4c NMDS analysis of sulfur cycle functional microbial communities demonstrated significant disparities between moderate expansion and no expansion (*p* = 0.045), mild expansion and no expansion (*p* = 0.0064), and HI and no expansion (*p* = 0.014) in the NMDS Score 2 marginal boxplot.

### 3.3. Shrub Expansion Effects on Microbial Community Composition

**Figure 5:**
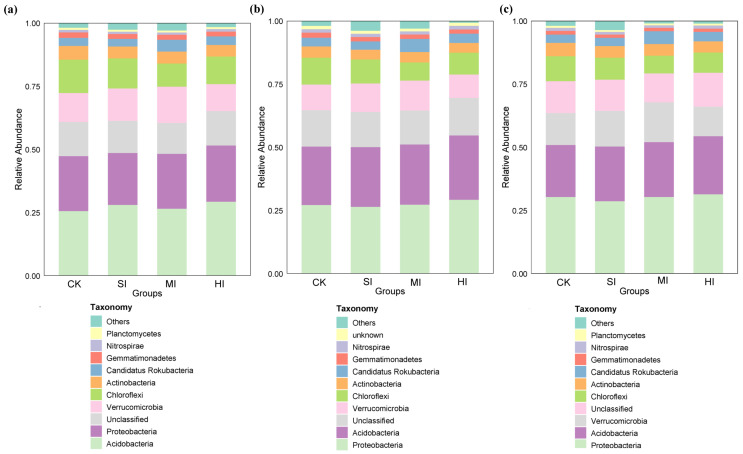
Differences in the composition of microbial communities involved in the cycle under different levels of shrub expansion. The composition of soil involved in the C cycle (**a**), N cycle (**b**), and S cycle (**c**) at the phylum level. CK: no expansion; SI: mild expansion; MI: moderate expansion; HI: severe expansion.

The expansion of shrubbery resulted in alterations to the composition of microbial communities associated with various cycles. However, these changes were observed to be similar across all levels of shrub expansion. The predominant microbial phyla within the microbial communities were identified as Acidobacteria, Proteobacteria, Verrucomicrobia, Chloroflexi, Actinobacteria, Candidatus Rokubacteria and Gemmatimonadetes (Figure 5). The relative abundance of Acidobacteria was highest in carbon cycle-related functional microbial communities (Figure 5a), while the relative abundance of Proteobacteria was highest in nitrogen and sulfur cycle-related functional microbial communities (Figure 5b,c). It is noteworthy that the alterations in Verrucomicrobia and Chloroflexi were uniform across all cycle-associated functional microbial communities. The relative abundance of Verrucomicrobia exhibited an initial increase with increasing shrub cover, subsequently decreasing, while the relative abundance of Chloroflexi demonstrated an initial decrease followed by an increase.

**Figure 6:**
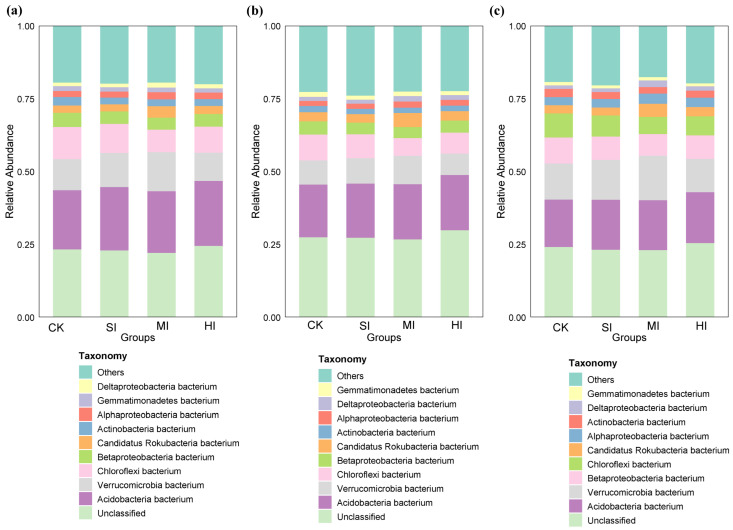
Differences in the composition of microbial communities involved in the cycle under different levels of shrub expansion. The composition of soil involved in the C cycle (**a**), N cycle (**b**), and S cycle (**c**) at the species level. CK: no expansion; SI: mild expansion; MI: moderate expansion; HI: severe expansion.

At the species level, the dominant species of cyclic functional microorganisms at sampling points with different degrees of shrub expansion were Acidobacteria bacterium, Verrucomicrobia bacterium, Chloroflexi bacterium, Betaproteobacteria bacterium, Candidatus Rokubacteria bacterium, and Actinobacteria bacterium. Notably, the relative abundance of Verrucomicrobia bacterium and Candidatus Rokubacteria bacterium among the various cyclic functional microorganisms showed consistent changes, first increasing and then decreasing as shrub expansion increased (Figure 6).

### 3.4. Carbon, Nitrogen, and Sulfur Metabolism: Metabolic Pathways and Genetic Changes

**Figure 7:**
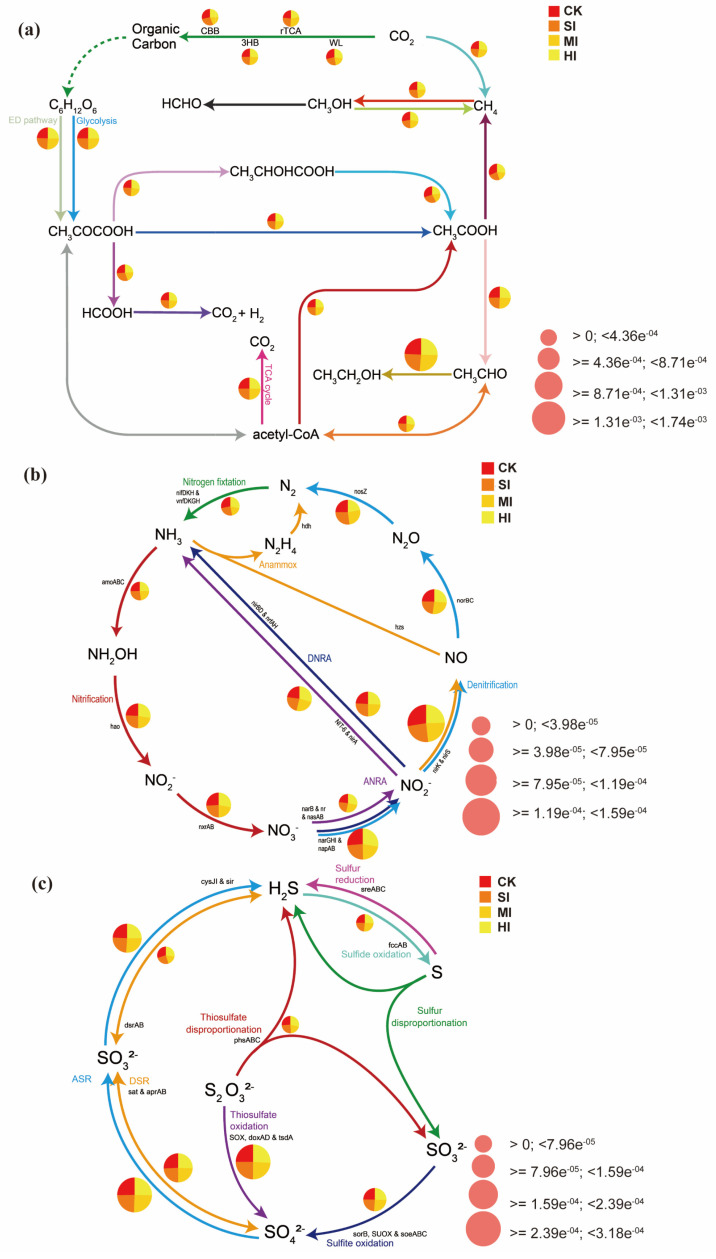
Main pathways of C (**a**), N (**b**), and S (**c**) metabolism. Each pie chart represents the composition of functional pathway abundance corresponding to different subgroups, and the size of the pie chart represents the total abundance size of each pathway. CK: no expansion; SI: mild expansion; MI: moderate expansion; HI: severe expansion.

We utilized data on the relative abundances of pathways involved in carbon, nitrogen, and sulfur cycles to calculate normalized relative abundances and created a visual cycle pathway diagram (Figure 7) to compare the relative abundances of biogeochemical pathways under different levels of shrub expansion. As shown in Figure 6a, shrub expansion reduces the abundance of the lactate-to-acetate conversion pathway, and the abundance of the acetate-dependent methane production pathway decreases significantly with increasing shrub expansion. Additionally, increasing shrub expansion significantly reduces the methane production process in which carbon dioxide is converted to methane during methane metabolism. As shrub expansion increases, the abundance of the anammox pathway, which reduces nitrate to nitrite (NO_3_^−^→NO_2_^−^) in nitrogen metabolism, decreases, and the abundance of the denitrification pathway, which converts nitrite to nitric oxide (nirK or nirS), also decreases (Figure 7b). In the sulfur cycle pathway diagram (Figure 7c), shrub expansion leads to a significant increase in the abundance of the pathway for the oxidation of thiosulfate to sulfate.

**Figure 8:**
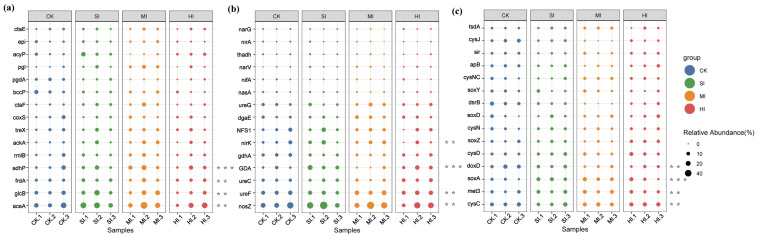
Gene bubble chart showing significant changes in the top 15 abundances. Genes involved in the C (**a**), N (**b**), and S (**c**) cycles. The size of the bubbles represents abundance. *** *p* < 0.001; ** *p* < 0.01; * *p* < 0.05 (based on Bootstrap 95% confidence intervals). CK: no expansion; SI: mild expansion; MI: moderate expansion; HI: severe expansion.

We identified the 15 genes with the highest abundance that showed the most significant changes in abundance among the genes associated with various cycles, standardized them, and visualized their changes using a bubble chart. Among the genes associated with the carbon cycle, aceA, glcB, frdA, and adhP showed the most significant changes due to shrub expansion (Figure 8a). Among nitrogen cycle-related genes, nosZ, ureF, GDA, and nirK showed the most significant changes due to shrub expansion. Notably, as the extent of shrub expansion increased, the abundance of nirK decreased significantly (Figure 8b). Among sulfur cycle-related genes, cysC, met3, soxA, and doxD showed the most significant changes due to shrub expansion (Figure 8c).

### 3.5. Correlation Analysis

**Figure 9:**
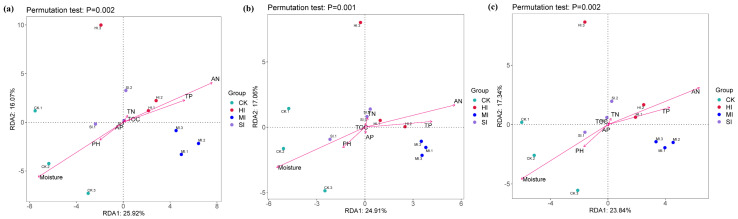
Redundancy analysis (RDA) of soil microorganisms participating in the carbon cycle (**a**), nitrogen cycle (**b**), and sulfur cycle (**c**) based on analyzed soil physical and chemical properties. CK, no expansion, SI; mild expansion, MI, moderate expansion. HI, severe expansion.

Redundancy analysis was used to identify the key drivers underlying changes in carbon, nitrogen, and sulfur cycles, as well as differences in the structure and composition of functional microbial communities. We found that moisture, AN, and TP were the primary environmental factors driving differences in the structure and composition of functional microbial communities associated with carbon, nitrogen, and sulfur cycles (Figure 9). Moisture was significantly positively correlated with no expansion but significantly negatively correlated with moderate expansion. AN and TP significantly negatively influenced the functional microbial communities at the CK level.

**Figure 10:**
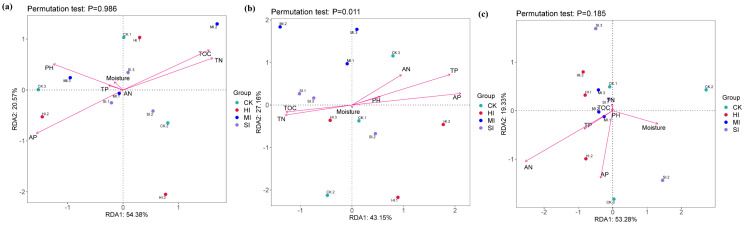
Based on the analysis of soil physical and chemical properties, redundancy analysis (RDA) was performed on genes involved in carbon cycles (**a**), nitrogen cycles (**b**), and sulfur cycles (**c**) pathways. CK, no expansion, SI; mild expansion, MI, moderate expansion. HI, severe expansion.

Based on an analysis of soil physical and chemical properties, the results of a redundancy analysis (RDA) conducted on genes involved in the carbon cycle (a), nitrogen cycle (b), and sulfur cycle (c) pathways. The results indicate that environmental factors are not the direct drivers of changes in carbon and sulfur cycle pathways (Figure 10a,c), while for the nitrogen cycle, TP and AP are the primary environmental factors driving changes in its pathways (Figure 10b). Environmental variables collectively explain 70.31% of the variation in gene composition, and the overall model is significantly validated through permutation tests.

**Figure 11:**
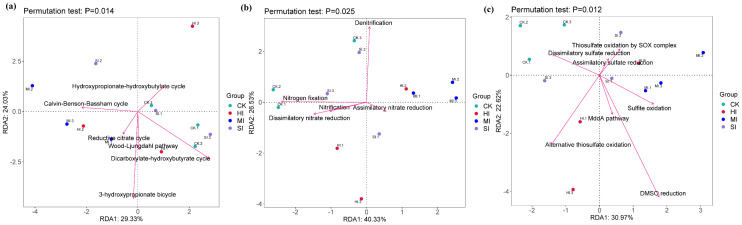
Based on the analysis of soil microorganisms, redundancy analysis (RDA) was performed on genes involved in carbon cycles (**a**), nitrogen cycles (**b**), and sulfur cycles (**c**) pathways. CK, no expansion, SI; mild expansion, MI, moderate expansion. HI, severe expansion.

As shown in Figure 11, changes in the 3-hydroxypropionic acid cycle caused by shrub expansion are the main factors explaining differences in the structure and composition of functional microbial communities in the carbon cycle. Denitrification and nitrogen fixation are the main factors explaining differences in the structure and composition of functional microbial communities in the nitrogen cycle, while dimethyl sulfide reduction is the main factor explaining differences in the structure and composition of functional microbial communities in the sulfur cycle.

## 4. Discussion

### 4.1. Shrub Expansion Significantly Altered Soil Properties

The present study found that shrub expansion significantly increased soil AN content while reducing soil moisture (Table 1). Soil moisture levels in mildly (SI) and moderately (MI) expanded areas were found to be significantly higher than in areas that had undergone more extensive expansion (HI), although these levels were still significantly lower than in the unexpanded control area (CK). The expansion of shrub vegetation has been demonstrated to exert a direct or indirect influence on the characteristics of soil moisture by means of altering the structure of vegetation communities and the texture of the soil [40]. This finding is in accordance with the classical theory of shrub expansion in arid ecosystems, which posits that the presence of deep-rooted shrubs leads to an exacerbation of soil drying through the processes of transpiration and root hydraulic redistribution [41]. It is, therefore, hypothesized that the increase in AN may be attributable to the ‘oasis effect’, which is formed by the accumulation of litter under the shrub canopy and the enhancement of rhizosphere microbial mineralization [42]. It is noteworthy that the short-term expansion of shrub vegetation did not result in significant alterations to the pH, total nitrogen (TN), total organic carbon (TOC), total phosphorus (TP), or available phosphorus (AP) levels. This observation indicates that these physicochemical properties demonstrate a delayed response to changes in vegetation. This finding lends further credence to the hypothesis that the ecological effects of shrub expansion exhibit temporal characteristics, with the water and nitrogen cycles responding initially, and the carbon and phosphorus pools requiring prolonged accumulation to undergo substantial changes [43,44]. The findings of this study suggest that alterations in soil nutrient and moisture content, resulting from increased shrub expansion, indicate that shrub expansion plays a pivotal role in regulating the material cycle processes within marsh soils.

### 4.2. Shrub Expansion Impacts on Microbial Community Diversity

The effects of shrub expansion on carbon, nitrogen, and sulfur cycle functional microbial communities exhibit a multidimensional response pattern. Despite the Shannon and Heip indices demonstrating no substantial disparities across varying expansion levels (see Figure 3a–c,g–i), suggesting stability in the uniformity and diversity of microbial communities, a notable increase in the Chao 1 index for carbon cycle functional microorganisms under moderate (MI) and severe (HI) expansion (see Figure 3d) indicates that augmented shrub cover may foster the proliferation of specific taxonomic units implicated in carbon transformation through the input of litter and root exudates. This phenomenon is consistent with the microbial mechanisms by which shrub expansion in temperate grasslands promotes organic matter transformation [45]. The influence of shrub expansion on diversity indices may be indirect, arising from alterations in the proportions of specific microbial functional groups (e.g., decomposers or symbionts). However, these non-significant trends may obscure potential responses in microbial communities [46]. It is noteworthy that the Chao 1 index for nitrogen/sulfur cycle microorganisms exhibited no significant alterations (Figure 3e,f), while the results of β-diversity analysis demonstrated that the community structure of all cycle microorganisms underwent a substantial transformation (Figure 4). This phenomenon underscores the functional redundancy of microbial ecosystems, where key ecological functions are maintained by similar functional groups even when community composition changes [47]. Microbial groups may engage in competition for the same resources through multi-species competition, thereby maintaining local α diversity stability through functional complementarity. However, community structure undergoes reorganization in response to environmental stress. Dengzeng et al. (2022) [48] found in a study of freshwater marsh shrub expansion that microbial β diversity significantly increased during the early stages of expansion, while α diversity significantly increased in later stages due to increased input of woody litter. These findings are consistent with those of this study.

### 4.3. Shrub Expansion Impacts on Microbial Community Composition

At the phylum level, Acidobacteria consistently dominated carbon cycle communities, while Proteobacteria dominated nitrogen/sulfur cycle communities (Figure 5). This stability is indicative of the core functional microbial communities’ capacity to resist disruption from habitat changes [49]. The abundance of Verrucomicrobia increased during mild expansion (SI), potentially benefiting from new carbon sources introduced by shrubs. However, during high-intensity expansion (HI), the abundance of Verrucomicrobia declined due to intensified resource competition. However, the initial decline in Chloroflexi abundance, which was followed by a recovery as shrub expansion intensified, suggests an ecological niche reconstruction strategy following high disturbance [50]. At the species level, the synchronous changes in Candidatus Rokubacteria and Verrucomicrobia (Figure 6) further indicate that soil organic matter input following shrub expansion provides microorganisms with abundant substrate resources, leading to significant proliferation [51], but declines due to intensified resource competition during high-intensity expansion (HI). The findings of this study lend support to the theory of microbial succession, which states that the effects of this process are delayed. The results demonstrate that in the short term, the expansion of shrubbery is the primary driver of community structure reorganization (β diversity) and fluctuations in the abundance of specific functional microorganisms. However, overall α diversity and core phylum composition remain resilient until the ecosystem reaches a threshold and enters a new steady state [52]. This finding is consistent with the results of the present study, which demonstrated that soil AN and moisture content exhibited rapid responses, while carbon and phosphorus pools demonstrated delayed responses.

### 4.4. Effects of Shrub Expansion on Soil Carbon, Nitrogen, and Sulfur Cycles

It was determined that shrub expansion had a significant impact on the cycles of carbon, nitrogen, and sulfur within the soil environment, as evidenced by the analysis of functional pathways and the dynamics of gene abundance. In the context of the carbon cycle, the observed decline in the abundance of the lactic acid → acetic acid pathway and the acetic acid-dependent methane production pathway (see Figure 7a) is indicative of a synergistic inhibition of fermentation processes and methane production. This inhibition is further supported by the downregulation of the key genes frdA and adhP (see Figure 8a). This phenomenon is associated with increased soil micro-domain oxygen flux [26], consistent with previous findings that shrub expansion regulates the production of carbon cycle substrates through a synergistic pathway involving “soil physicochemical-functional genes.” The comprehensive decline in denitrification pathways (NO_3_^−^ → NO_2_^−^; NO_2_^−^ → NO) (Figure 7b) is directly driven by the synchronous downregulation of the nirK and nosZ genes (Figure 8b). It is noteworthy that the increased abundance of ureF suggests a compensatory enhancement of the urea hydrolysis pathway, which may represent an adaptive response to shifts in nitrogen storage forms following increased shrub litter input [53]. In the sulfur cycle, the oxidation of thiosulfate (S_2_O_3_^2−^) to sulfate (SO_4_^2−^) is a core step in sulfur transformation, and shrub expansion significantly increased the abundance of this pathway (Figure 7c). Concurrently, pivotal sulfur cycle genes cysC, met3, soxA, and doxD exhibit a rapid response to shrub expansion (Figure 8c), a phenomenon that may be attributable to the enhanced adaptability of sulfur-oxidizing bacterial communities to fluctuations in moisture gradients triggered by shrub expansion [54,55]. In summary, the coupled changes in carbon, nitrogen, and sulfur cycles indicate that shrub expansion disrupts the chemical balance between elements by reshaping microbial functional networks, which may further affect greenhouse gas fluxes and nutrient retention capacity in wetland ecosystems [56].

### 4.5. Correlation Analysis

The present study combined environmental driver analysis and redundancy analysis (RDA) to reveal the hierarchical regulatory mechanisms underlying functional microbial community restructuring under shrub expansion. Collectively, moisture, AN, and TP dominated the overall community structure variability of microorganisms involved in carbon, nitrogen, and sulfur cycles (Figure 9), confirming the synergistic screening effect of water stress and nutrient availability on microbial assembly [57]. However, it is important to note that the regulation of functional pathways by environmental factors exhibits specificity across different cycles. Changes in metabolic pathways associated with the carbon and sulfur cycles (see Figure 10) were not found to be significantly correlated with environmental factors. This suggests that certain microorganisms may have served to mitigate the effects of environmental fluctuations through metabolic complementarity [58]. In contrast, nitrogen cycle pathways are directly driven by TP and AP, a phenomenon that can be explained by the findings of Zeng et al. [59]. This is due to the fact that phosphorus imposes rigid constraints on the activity of nitrogenase (nifH) and denitrification enzymes (narG). The P cycle and its functional microorganisms’ response to shrub expansion will be discussed in greater detail in subsequent studies.

At the metabolic functional level, correlation analyses were conducted between various cycle pathways and functional microorganisms, revealing that shrub expansion specifically activates key pathways in the C, N, and S cycles (Figure 11). Changes in the abundance of carbon cycle pathways are primarily driven by the 3-hydroxypropionate bicycle, which has been shown to be executed by Chloroflexi, efficiently fixing CO_2_ through carboxylation of propionyl-CoA [60]. Changes in the abundance of nitrogen cycle pathways are jointly regulated by denitrification and nitrogen fixation. It is hypothesized that this is primarily due to the functional antagonism between Bradyrhizobium (nitrogen-fixing bacteria) and Pseudomonas (denitrifying bacteria) within the Proteobacteria phylum, which jointly regulate the dynamic balance between nitrogen loss and input, consistent with the findings of Yang et al. [61] The sulfur cycle is dependent on the DMSO reduction pathway. Research has indicated that this process is mediated by the Rhodobacter genus of Proteobacteria, which reduces dimethyl sulfoxide to volatile sulfides [62]. Furthermore, the sensitivity of the aforementioned process may amplify the moisture response effects of sulfur-oxidizing bacteria [63]. Environmental factors influence changes in soil microbial community structure but do not directly affect elemental cycle pathways. This observation highlights the complex functions of microorganisms in responding to disturbances and underscores the complexity of ecological dynamics.

Therefore, the expansion of temperate wetland shrubs triggers a soil-microbial-cycle cascade effect that reshapes microbial communities. Functional redundancy buffers carbon/nitrogen cycles from environmental fluctuations, while obligate species exacerbate the sensitivity of nitrogen cycles to phosphorus limitations, and compensatory pathways cannot fully offset element losses. This mechanistic framework explains why wetlands invaded by shrubs experience a significant reduction in carbon storage despite temporary microbial resilience.

## 5. Conclusions

Although α diversity remained largely unaffected, with the exception of the Chao 1 index of carbon cycle-related functional microorganisms, β diversity of functional microbial communities within each cycle exhibited significant alterations. The abundance of various cycle pathways and their associated functional genes exhibited clear alterations. It is evident that the primary factors influencing soil carbon, nitrogen, and sulfur cycles, as well as the structure and metabolism of functional microbial communities, are ammonia nitrogen, moisture, and total phosphorus. The alterations in the prevalence of processes such as the 3-hydroxypropionate cycle, denitrification, nitrogen fixation, and DMSO reduction, resulting from shrub expansion, are the primary factors elucidating the disparities in the structure and composition of functional microbial communities involved in carbon, nitrogen, and sulfur cycles. Future research will focus on more precisely quantifying the direct and indirect effects of these factors, elucidating how shrub expansion influences wetland carbon, nitrogen, and sulfur cycles as well as functional microbial processes, and clarifying their regulatory mechanisms. For example, the PLS-PM method was used to integrate metabolomics and transcriptomics with ^13^C tracing technology to reveal the mechanism of gene–enzyme–metabolite cascade reactions in soil microbial metabolism driven by shrub expansion. The findings of this study are expected to provide a theoretical basis for the management of shrub expansion and wetland functional assessments and to offer scientific support for global wetland conservation.

## Figures and Tables

**Table 1:** Physicochemical properties of soils with different levels of shrub expansion.

Plot	PH	TN (g/kg)	TOC (g/kg)	TP (g/kg)	AP (mg/kg)	AN (mg/kg)	Moisture (%)
CK	5.35 ± 0.12 a	3.00 ± 0.24 a	33.48 ± 3.87 a	0.84 ± 0.07 a	8.75 ± 1.62 a	0.24 ± 0.03 b	81.54 ± 0.98 a
SI	5.22 ± 0.12 a	3.75 ± 0.84 a	39.85 ± 8.91 a	1.03 ± 0.15 a	8.26 ± 4.14 a	0.31 ± 0.01 a	71.20 ± 0.13 b
MI	5.26 ± 0.14 a	3.27 ± 1.65 a	35.42 ± 19.41 a	1.07 ± 0.17 a	8.17 ± 2.92 a	0.32 ± 0.02 a	70.67 ± 0.46 b
HI	5.20 ± 0.12 a	3.31 ± 1.20 a	34.44 ± 13.59 a	1.09 ± 0.10 a	10.17 ± 3.88 a	0.34 ± 0.02 a	68.88 ± 0.59 c

Values are presented as the mean ± standard error (*n* = 3). Different letters indicate significant differences between treatments (*p* < 0.05). Abbreviations: TN, total nitrogen; TOC, total organic carbon; TP, total phosphorus; AP, available phosphorus; AN, ammonia nitrogen. CK: no expansion; SI: mild expansion; MI: moderate expansion; HI: severe expansion.

## Data Availability

The original contributions presented in this study are included in the article. Further inquiries can be directed to the corresponding authors.

## References

[1] Chen Z., Zhang C., Liu Z., Song C., Xin S. (2023). Effects of Long-Term (17 Years) Nitrogen Input on Soil Bacterial Community in Sanjiang Plain: The Largest Marsh Wetland in China. Microorganisms.

[2] Liao Q., Lu C., Yuan F., Fan Q., Chen H., Yang L., Qiu P., Feng Z., Wang C., Zou X. (2023). Soil carbon-fixing bacterial communities respond to plant community change in coastal salt marsh wetlands. Appl. Soil Ecol..

[3] Saunois M., Stavert A.R., Poulter B., Bousquet P., Canadell J.G., Jackson R.B., Raymond P.A., Dlugokencky E.J., Houweling S., Patra P.K. (2020). The Global Methane Budget 2000–2017. Earth Syst. Sci..

[4] Poulter B., Bousquet P., Canadell J.G., Ciais P., Peregon A., Saunois M., Arora V.K., Beerling D.J., Brovkin V., Jones C.D. (2017). Global wetland contribution to 2000–2012 atmospheric methane growth rate dynamics. Environ. Res. Lett..

[5] Huang Y., Sun W., Zhang W., Yu Y., Su Y., Song C. (2010). Marshland conversion to cropland in northeast China from 1950 to 2000 reduced the greenhouse effect. Glob. Change Biol..

[6] Wang Z.M., Zhang B., Zhang S.Q., Li X.Y., Liu D.W., Song K.S., Li J., Li F., Duan H. (2006). Changes of land use and of ecosystem service values in Sanjiang Plain, Northeast China. Environ. Monit. Assess..

[7] Shen X., Liu B., Jiang M., Lu X. (2020). Marshland loss warms local land surface temperature in China. Geophys. Res. Lett..

[8] Mao D., He X., Wang Z., Tian Y., Xiang H., Yu H., Man W., Jia M., Ren C., Zheng H. (2019). Diverse policies leading to contrasting impacts on land cover and ecosystem services in Northeast China. J. Clean. Prod..

[9] Wang Z., Mao D., Li L., Jia M., Dong Z., Miao Z., Ren C., Song C. (2015). Quantifying changes in multiple ecosystem services during 1992–2012 in the Sanjiang Plain of China. Sci. Total Environ..

[10] Yan F.Q., Zhang S.W. (2019). Ecosystem service decline in response to wetland loss in the Sanjiang Plain, Northeast China. Ecol. Eng..

[11] Phillips C.A., Wurzburger N. (2019). Elevated rates of heterotrophic respiration in shrub-conditioned arctic tundra soils. Pedobiologia.

[12] Broadbent Arthur A.D., Michael B., Pritchard William J., Newbold Lindsay K., Tim G., Andrew G., Snell Helen S.K., Irene C., Antonios M., Grant Helen K. (2021). Shrub expansion modulates belowground impacts of changing snow conditions in alpine grasslands. Ecol. Lett..

[13] Oriol G., Karita S., Ninot Josep M., József G., Annamari M., Ahonen Saija H.K., Josep P. (2019). Encroachment of shrubs into subalpine grasslands in the Pyrenees modifies the structure of soil fungal communities and soil properties. FEMS Microbiol. Ecol..

[14] Frey B., Carnol M., Dharmarajah A., Brunner I., Schleppi P. (2020). Only minor changes in the soil microbiome of a sub-alpine forest after 20 years of moderately increased nitrogen loads. Front. For. Glob. Change.

[15] Basu S., Kumar G., Chhabra S., Prasad R. (2021). Role of soil microbes in biogeochemical cycle for enhancing soil fertility. New and Future Developments in Microbial Biotechnology and Bioengineering.

[16] Lakshmi G., Okafor B.N., Visconti D. (2020). Soil microarthropods and nutrient cycling. Environment, Climate, Plant and Vegetation Growth.

[17] Wang Y., Wu F., Li X., Li C., Zhao Y., Gao Y., Liu J. (2023). Effects of plants and soil microorganisms on organic carbon and the relationship between carbon and nitrogen in constructed wetlands. Environ. Sci. Pollut. Res..

[18] Sun Y., Zhang Y., Feng W., Qin S., Liu Z., Bai Y., Yan R., Fa K. (2017). Effects of xeric shrubs on soil microbial communities in a desert in northern China. Plant Soil.

[19] Zhang T., Ma W., Tian Y., Bai S., Zuoma D., Zhang D., Ma X., Mu X. (2023). The mitigation of microbial carbon and nitrogen limitations by shrub encroachment: Extracellular enzyme stoichiometry of the alpine grassland on the Qinghai-Tibetan Plateau. Biogeochemistry.

[20] Bragazza L., Bardgett R.D., Mitchell E.A.D., Buttler A. (2015). Linking soil microbial communities to vascular plant abundance along a climate gradient. New Phytol..

[21] Fan Y., Li X.Y., Huang H., Wu X.C., Yu K., Wei J.Q., Zhang C., Wang P., Hu X., D’Odorico P. (2019). Does phenology play a role in the feedbacks underlying shrub encroachment?. Sci. Total Environ..

[22] Van Der Heijden M.G.A., Bardgett R.D., Van Straalen N.M. (2008). The unseen majority: Soil microbes as drivers of plant diversity and productivity in terrestrial ecosystems. Ecol. Lett..

[23] Petrie M.D., Collins S.L., Swann A.M., Ford P.L., Litvak M. (2015). Grassland to shrubland state transitions enhance carbon sequestration in the northern Chihuahuan Desert. Glob. Change Biol..

[24] Zhao J., Yang W., Ji-Shi A., Ma Y., Tian L., Li R., Huang Z., Liu Y.-F., Leite P.A., Ding L. (2023). Shrub encroachment increases soil carbon and nitrogen stocks in alpine grassland ecosystems of the central Tibetan Plateau. Geoderma.

[25] Mauclet E., Agnan Y., Hirst C., Monhonval A., Pereira B., Vandeuren A., Villani M., Ledman J., Taylor M., Jasinski B.L. (2022). Changing sub-Arctic tundra vegetation upon permafrost degradation: Impact on foliar mineral element cycling. Biogeosci. Discuss..

[26] Wang W., Kang Y., Liu M., Yang T., Bao D., Ji W., Su J. (2025). Shrub encroachment in alpine meadows facilitates soil carbon and nitrogen storage by redistributing soil water. Catena.

[27] Mack M.C. (2016). Collaborative Research: Shrub Impacts on Nitrogen Inputs and Turnover in the Arctic, and the Potential Feedbacks to Vegetation and Climate Change. NSF Award Number 1556496.

[28] Weng X.H., Sui X., Li M.S., Liu Y.N., Zhang R.T., Yang L.B. (2022). Effects of Simulated Nitrogen Deposition on Soil Microbial CarbonC Metabolism in Calamagrostis angustifolia Wetland in Sanjiang Plain. Huan Jing Ke Xue.

[29] Zhang R., Fu X., Zhong H., Sui X., Liu Y. (2023). Changes in Soil Bacterial Community and Function in Winter Following Long-Term Nitrogen (N) Deposition in Wetland Soil in Sanjiang Plain, China. Microorganisms.

[30] Zhang R.T., Wang S.Z., Zhong H.X., Sui X., Liu Y.-N. (2025). Seasonal variations in the soil microbiota of a temperate wetland in Northeast China in response to nitrogen deposition. Catena.

[31] Hua Z.S., Han Y.J., Chen L.X., Liu J., Hu M., Li S.-J., Kuang J.-L., Chain P.S.G., Huang L.-N., Shu W.-S. (2015). Ecological roles of dominant and rare prokaryotes in acid mine drainage revealed by metagenomics and metatranscriptomics. ISME J..

[32] de Sena Brandine G., Smith A.D. (2021). Falco: High-speed FastQC emulation for quality control of sequencing data. F1000Research.

[33] Peng Y., Leung H.C., Yiu S.M., Chin F.Y.L. (2012). IDBA-UD: A de novo assembler for singlecell and metagenomic sequencing data with highly uneven depth. Bioinformatics.

[34] Huson D.H., Auch A.F., Qi J., Schuster S.C. (2007). MEGAN analysis of metagenomic data. Genome Res..

[35] Kanehisa M., Furumichi M., Tanabe M., Sato Y., Morishima K. (2017). KEGG: New perspectives on genomes, pathways, diseases and drugs. Nucleic Acids Res..

[36] Buchfink B., Xie C., Huson D.H. (2015). Fast and sensitive protein alignment using DIAMOND. Nat. Methods.

[37] Love M.I., Huber W., Anders S. (2014). Moderated estimation of fold change and dispersion for RNA-seq data with DESeq2. Genome Biol..

[38] Wickham H., Bryan J. (2023). readxl: Read Excel Files (Version 1.4.3) [R Package]. Comprehensive R Archive Network. https://CRAN.R-project.org/package=readxl.

[39] Hester J. (2023). glue: Interpreted String Literals (Version 1.6.2) [R Package]. Comprehensive R Archive Network. https://CRAN.R-project.org/package=glue.

[40] Cui Z., Wu G.L., Huang Z., Liu Y. (2019). Fine roots determine soil infiltration potential than soil water content in semi-arid grassland soils. J. Hydrol..

[41] Richards J.H., Caldwell M.M. (1987). Hydraulic lift: Substantial nocturnal water transport between soil layers by Artemisia tridentata roots. Oecologia.

[42] Schlesinger W.H., Raikes J.A., Hartley A.E., Cross A.F. (1996). On the spatial pattern of soil nutrients in desert ecosystems. Ecology.

[43] Eldridge D.J., Bowker M.A., Maestre F.T., Roger E., Reynolds J.F., Whitford W.G. (2011). Impacts of shrub encroachment on ecosystem structure and functioning: Towards a global synthesis. Ecol. Lett..

[44] D’Odorico P., Okin G.S., Bestelmeyer B.T. (2012). A synthetic review of feedbacks and drivers of shrub encroachment in arid grasslands. Ecohydrology.

[45] Du Z., Zheng H., Penuelas J., Sardans J., Deng D., Cai X., Gao D., Nie S., He Y., Lü X. (2024). Shrub encroachment leads to accumulation of C, N, and P in grassland soils and alters C: N: P stoichiometry: A meta-analysis. Sci. Total Environ..

[46] Wang S.K., Zuo X.A., Zhao X.Y., Li Y.-Q., Zhou X., Lv P., Luo Y.-Q., Yun J.-Y. (2016). Responses of soil fungal community to the sandy grassland restoration in Horqin Sandy Land, northern China. Environ. Monit. Assess..

[47] Louca S., Polz M.F., Mazel F., Albright M.B., Huber J.A., O’Connor M.I., Ackermann M., Hahn A.S., Srivastava D.S., Crowe S.A. (2018). Function and functional redundancy in microbial systems. Nat. Ecol. Evol..

[48] Zhuoma D., Ma W., Wang C., Tang S., Zhang D. (2022). Effect of shrub encroachment on alpine grass soil microbial community assembly. Front. Soil Sci..

[49] Fierer N., Leff J.W., Adams B.J., Nielsen U.N., Bates S.T., Lauber C.L., Owens S., Gilbert J.A., Wall D.H., Caporaso J.G. (2012). Cross-biome metagenomic analyses of soil microbial communities and their functional attributes. Proc. Natl. Acad. Sci. USA.

[50] Debray R., Herbert R.A., Jaffe A.L., Crits-Christoph A., Power M.E., Koskella B. (2022). Priority effects in microbiome assembly. Nat. Rev. Microbiol..

[51] Agnolucci M., Battini F., Cristani C., Giovannetti M. (2015). Diverse bacterial communities are recruited on spores of different arbuscular mycorrhizal fungal isolates. Biol. Fertil. Soils.

[52] Fukami T. (2015). Historical contingency in community assembly: Integrating niches, species pools, and priority effects. Annu. Rev. Ecol. Evol. Syst..

[53] Hou S.L., Hättenschwiler S., Yang J.J., Sistla S., Wei H., Zhang Z., Hu Y., Wang R., Cui S., Lü X. (2021). Increasing rates of long-term nitrogen deposition consistently increased litter decomposition in a semi-arid grassland. New Phytol..

[54] Chen L., Zhang Q., Zhang W., Zhu K., Xu Q., Hu K., Feng Q., Liu Y., Wang B. (2025). Metagenomic insights into the effects of desiccation on functions associated with biogeochemical cycles and resistome of microbial communities. Appl. Soil Ecol..

[55] Rathour R., Gupta J., Mishra A., Rajeev A.C., Dupont C.L., Thakur I.S. (2020). A comparative metagenomic study reveals microbial diversity and their role in the biogeochemical cycling of Pangong lake. Sci. Total Environ..

[56] Schlesinger W.H., Bernhardt E.S. (2013). Biogeochemistry: An Analysis of Global Change.

[57] Li Y., Wang J., Li E., Yang X., Yang J. (2024). Shifts in Microbial Community Structure and Co-occurrence Network along a Wide Soil Salinity Gradient. Microorganisms.

[58] Allison S.D., Martiny J.B.H. (2008). Resistance, resilience, and redundancy in microbial communities. Proc. Natl. Acad. Sci. USA.

[59] Zeng Q., Zhang Q., Fan Y., Gao Y., Yuan X., Zhou J., Dai H., Chen Y. (2024). Phosphorus availability regulates nitrogen fixation rate through a key diazotrophic assembly: Evidence from a subtropical Moso bamboo forest subjected to nitrogen application. Sci. Total Environ..

[60] Alber B.E., Fuchs G. (2002). Propionyl-coenzyme A synthase from *Chloroflexus aurantiacus*, a key enzyme of the 3-hydroxypropionate cycle for autotrophic CO_2_ fixation. J. Biol. Chem..

[61] Yang X., Tang S., Ni K., Shi Y., Yi X., Ma Q., Cai Y., Ma L., Ruan J. (2023). Long-term nitrogen addition increases denitrification potential and functional gene abundance and changes denitrifying communities in acidic tea plantation soil. Environ. Res..

[62] Zinder S.H., Brock T.D. (1978). Dimethyl sulfoxide as an electron acceptor for anaerobic growth. Arch. Microbiol..

[63] Wu B., Liu F., Fang W., Yang T., Chen G.-H., He Z., Wang S. (2021). Microbial sulfur metabolism and environmental implications. Sci. Total Environ..

